# The complete mitochondrial genome of Antarctic *Phaeodactylum tricornutum* ICE-H

**DOI:** 10.1080/23802359.2020.1788441

**Published:** 2020-07-11

**Authors:** Lina Liu, Yingying He, Kai Wang, Jinlai Miao, Zhou Zheng

**Affiliations:** a Department of Specialty Medicine, School of Basic Medicine, Qingdao University, Qingdao, China; b Key Laboratory of Marine Bioactive Substances, First Institute of Oceanography, Ministry of Natural Resource, Qingdao, China; c Laboratory for Marine Drugs and Bioproducts of Qingdao National Laboratory for Marine Science and Technology, Qingdao, China

**Keywords:** Mitochondrial genome, *Phaeodactylum tricornutum* ICE-H, Antarctica

## Abstract

In this study, the complete mitochondrial genome (mtDNA) of the Antarctic *Phaeodactylum tricornutum* ICE-H was sequenced using Illumina NovaSeq PE150. The circular mtDNA was 77055 bp in size and encodes 60 genes, contains 24 tRNA genes, 34 protein-coding genes, and 2 rRNA genes. The composition of A + T in ICE-H mtDNA was 65.34%. The phylogenetic relationship of 17 species of plant mitochondria were analyzed using the maximum likelihood method by the MEGA-X. *Phaeodactylum tricornutum* ICE-H was most closely related to *Phaeodactylum tricornutum*.

Antarctic sea ice microalgae have crucial effects in maintaining the stability of polar ecosystems (Thomas and Dieckmann 2002). They contain many active substances and have great potential to be used as biotechnology products (Zuliani et al. 2016). *Phaeodactylum tricornutum* ICE-H belongs to Phaeodactylaceae, a eukaryotic alga that can grow in extreme Antarctic environment. Therefore, there is an urgent need to understand the genome information of ICE-H. Here, we first assemble and annotate the complete mitochondrial genome (mtDNA) of the Antarctic ICE-H.

Samples used for sequencing were isolated from floating ice near the Zhongshan Research Station of Antarctica (S 69°48′, E 77°48′). The sample (Accession no. FIO2008697701) is stored in the Key Laboratory of First Institute of Oceanography, Ministry of Natural Resources. We isolated the complete mtDNA of ICE-H and sequenced it based on the NovaSeq PE150 at the Beijing Novogene Bioinformatics Technology Co., Ltd. The mtDNA was assembled by SOAP denovo (version 2.04) (Li et al. 2008, 2010), integrated using CISA (Lin and Liao 2013) and finally forecasted by GeneMarkS (Version4.17) (Besemer et al. 2001).

The complete mtDNA of ICE-H is 77055 bp (GenBank registration number: MN956530.1), including 34 protein-coding genes, 24 tRNA genes, and 2 rRNA genes. The mtDNA of ICE-H composition is 33.40% for A, 16.71% for C, 17.95% for G, and 31.94% for T. The percentage of A + T in ICE-H mtDNA was 65.34%. The 34 PCGS include COX2-3, NAD1-7, NAD4l, NAD11-a, NAD11-b, RPL6, RPL2, RPL5, RPL14, RPL16, RPS2-4, RPS8, RPS10, RPS12-13, RPS19, ATP6, ATP8, ATP9, COB, TATC. Among the 34 PCGS, except NAD7, COB all protein initiation codons are ATG. The termination codon of most protein-coding genes was TAA (5 of 34 genes) or TAG (3 of 34 genes). The 24 tRNA-coding genes ranged in size from 72 bp to 93 bp.

Phylogenetic relationship of 17 complete mtDNAs was analyzed using the maximum likelihood method with 1000 bootstraps (Kumar et al. 2016), which revealed that the ICE-H were most affinitive to the *Phaeodactylum tricornutum* (HQ840789.1). And the distance between Oryza Sativa Indica Group and Zea mays subsp. was the farthest (Figure 1). The results of the study help to construct the molecular identification system of the Phaeodactylaceae and contribute to the phylogenetic research of the Bacillariophyta family.

**Figure 1.**
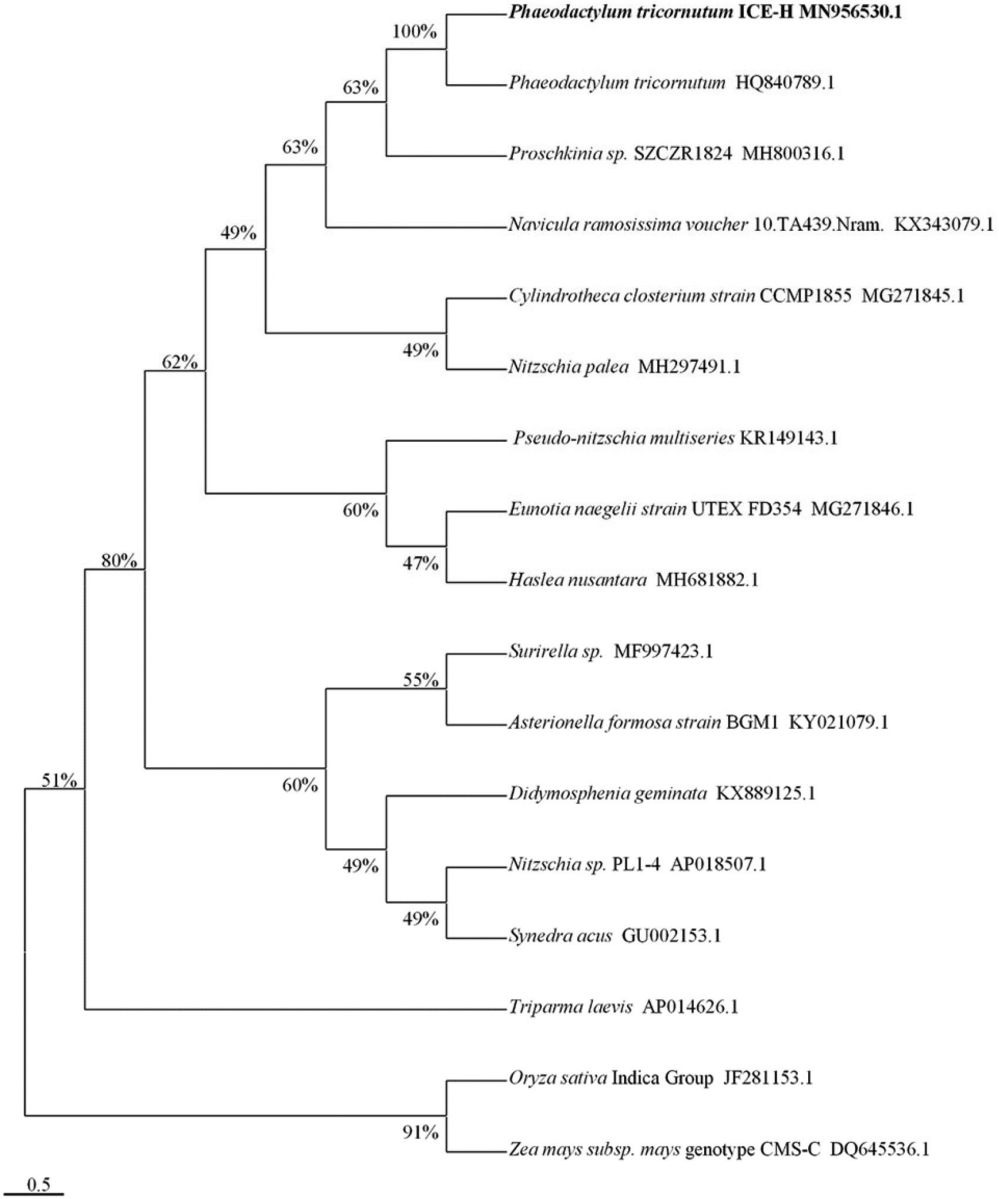
Maximum likelihood phylogenetic tree based on 17 complete mtDNAs.

## Data Availability

The data that support the findings of this study are openly available in NCBI (https://www.ncbi.nlm.nih.gov/) (GeneBank Number: MN956530.1).
